# Optimization of Flat Ends in Pressure Vessels

**DOI:** 10.3390/ma12244194

**Published:** 2019-12-13

**Authors:** Bogdan Szybiński, Paweł J. Romanowicz

**Affiliations:** Institute of Machine Design, Cracow University of Technology, ul. Warszawska 24, 31-155 Cracow, Poland; promek@mech.pk.edu.pl

**Keywords:** pressure vessels, flat ends, stress concentration, parametric optimization, topological optimization

## Abstract

The application of flat ends in pressure boilers is inevitably associated with the presence of stress concentration, which is observed in the vicinity of the junction of the cylinder and the closing flat plate. The analyzed flat end plates with stress relief grooves fall into the group of solutions recognized by the respective Standards of Calculations of Pressure Vessels. Unfortunately, no clear evidence is given in the Standards on how to choose the best groove parameters. This opens up the problem of the optimal choice of the groove parameters providing a minimum stress level. Even for the optimal values defining the stress relief groove geometry, certain plastic deformations are observed in the groove area for materials which exhibit elastic-plastic properties. Such a situation is completely unacceptable during exploitation, and a suitable reduction of the operating pressure is necessary. This paper discusses the effectiveness of other designs for flat ends used in pressure vessels. The proposed modifications took the form of external ribs applied around the top of the endplate circumference. The dimensions of these ribs were set using parametric optimization. The results of the study encouraged the authors to perform a more general analysis with the use of topology optimization. The results of all performed studies proved that the reduction of stress concentration and the full elimination of plastic deformation are possible. All numerical calculations were made using the finite element code (FEM), Ansys.

## 1. Introduction

In standard applications, commonly-used pressure vessels are closed by ellipsoidal or hemispherical heads. Their manufacturing process is not cheap, but is well established. The applications of such heads provide the minimum stress concentration in the vicinity of the junction between the cylindrical part of the vessel and the closure [1,2,3,4]. In certain situations (i.e., small internal diameters), flat endplates can also be alternatively applied, which are less complex in shape and much cheaper in fabrication. The main disadvantage of such a design is the presence of the severe stress concentration in the junction of the flat plate edge and the shell wall of the boiler. In order to reduce the notch effect, a stress relief groove can be applied, which provides a smoother shape transition in the shell-endplate junction and releases the stress concentration. Even in this case, due to the lack of continuity of the curvature along the contour in the area where the inner edge of the cylindrical wall transforms to the curved one, a certain stress concentration is still observed [5,6,7,8]. Effectively modeling the junction between the pressure boiler wall and the flat head of the boiler has been a challenge for researchers for many years [1,2,3,4,7,9]. Usually, the minimization of the stress peak is achieved by the introduction of respective functions approximating the curved shape of the shell, which provides continuity of the curvature on the inner skin of the vessel. However, this approach is still not commonly applied in the construction of flat heads of boilers due to manufacturing problems.

**Figure 1:**
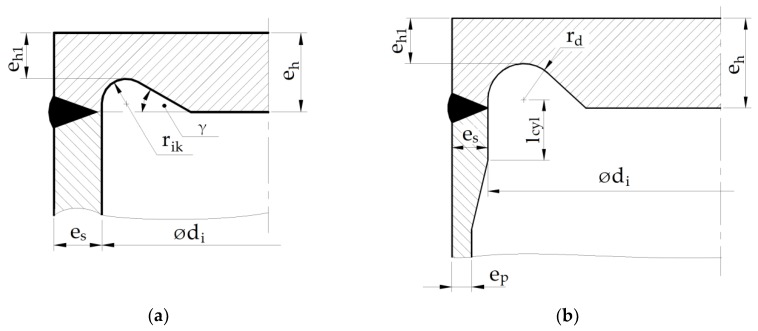
Two recommended designs for boilers with flat ends with stress relief grooves: (**a**) EN 12952:3 code; (**b**) EN 13445:3 code.

There are two commonly-used boiler codes for the calculation of pressure apparatuses (EN 12952:3 [10], EN 13445-3 [11]). Both suggest the application of a circular shape for the stress relief groove. However, the choice of the groove parameters is slightly different in each of them. The main differences between the approaches are illustrated in Figure 1. As shown, the suggested in EN 13445-3 change of the shape relies on the local thickness increase of the cylindrical shell wall in the vicinity of the shell–endplate connection. This results in a variation of admissible groove parameters calculated according to the respective code applied in boiler calculations. In previous studies [9,12,13,14], it was observed that the geometries of relieve grooves, acceptable from the point of view of the Standard EN 12952:3, may significantly differ in strength, durability, or other engineering parameters. Experimental tests were performed for the cylindrical pressure vessel with flat ends made from 16Mo3 steel with an external diameter ϕ406.4 mm [12]. The nominal pressure was set to *p_c_* = 22 MPa on the basis of the minimal measured wall thickness of the tube. Two kinds of flat ends with different shapes of stress relief grooves were applied in the experimental tests. Such geometries (optimal and nonoptimal with respect to the minimum value of the equivalent plastic strain) were selected after numerical optimization (finite element method). The obtained numerical and experimental results were compared and discussed in detail in the [12]. The performed numerical studies, verified by the experiments, revealed the presence of plastic zones which appeared in the cut-out regions. However, the range and the values of the equivalent plastic deformations varied depending on the groove geometry. The maximal value of the equivalent plastic strains for the nonoptimal configuration was several times higher than the respective one for the optimal groove configuration. Detailed results are given in [12]. The presence of such zones of plastic strains in the vicinities of the weld zones, in which material is also subjected to structural changes, may lead to a local increase in hardness and decrease in the fracture parameters in the weld and heat-affected zones [15]. In extreme cases (ductile material, the existence of internal cracks or voids, etc.), a high-stress concentration for the nonoptimal shape of the stress relief groove may lead to sudden, premature catastrophic failure. These factors justify the optimization of stress relief grooves in flat ends; this task has become an important engineering issue. The optimal choice of the structure parameters becomes particularly important in the case of boilers subjected to cyclic loadings, when the risk of premature failure exists. Such problems can be analyzed with the application of the fracture mechanics criteria, which were recently compared and discussed in [16]. 

The main aim of this paper is the optimization of the stress relief groove and flat end geometry with respect to the Standards EN 13445-3 in order to decrease or eliminate plastic deformation in pressure vessels. The above standard proposes a slight modification of the cylinder-end area, which relies on the introduction of a local increase of the pipe wall thickness. First, the effectiveness of such a geometry was evaluated. The performed numerical studies show the increase of the maximal equivalent plastic strain with a local increase in the wall thickness (Figure 1b). That was the reason why the boiler with a constant, basic wall thickness was chosen for further investigation. The introduced geometry modifications of the structures relied on the application of circumferential ribs placed along the outside boundary of the endplate. Two shapes of ribs were studied, i.e., with rectangular and circular cross-sections. The performed studies resulted in the full elimination of plastic deformations in the most strenuous area. Following these encouraging results, a more general approach was applied in the final analysis, i.e., structural topology optimization was performed. Such a technique was recently successfully used in optimization procedures for different engineering problems [17,18,19,20]. Various combinations of added material were investigated in the optimization of the structural topology. The application of such an approach also allowed to elimination of plastic deformations and reduction of stress concentration, with more convenient distribution of the material. 

The paper consists of five sections. The introduction to the investigated problems is described in Section 1. The fundamental theory concerning the design of a flat end with a stress relief groove according to standards is given in Section 2. The numerical model and description of the material are depicted in Section 3. The results of the optimization of flat ends are illustrated and discussed in Section 4. The conclusions are given in Section 5.

## 2. Stress relief Groove Design 

The starting point for calculations of flat ends with a stress relief groove was the assessment of the endplate thickness, which is calculated using the following formula:

(1) $$e_{h}=C_{1}\cdot C_{2}\cdot C_{3}\cdot d_{i}\cdot \sqrt{\frac{p_{c}}{f}}.$$

The above equation is valid for the code EN 12952:3. In Equation (1), *d_i_* is the internal diameter of the boiler, constants *C_2_* and *C_3_* are set to 1.0 (for boilers with circular cross-sections and endplates without openings), and constant *C_1_* depends on the ratio between applied pressure *p_c_* and the admissible stress *f* applied in structure calculations. As a result, *C_1_* varies between 0.41 and 0.82, and is taken from the plot included in the code. In the case of the code EN 13445-3, the endplate thickness is calculated as follows:

(2) $$e_{h}=\max\left\{\begin{array}{cc} C_{1}d_{i}\cdot \sqrt{\frac{p_{c}}{f}}, & C_{2}d_{i}\cdot \sqrt{\frac{p_{c}}{f_{\min}}} \end{array}\right\},$$

where the *d_i_* is the inner boiler diameter measured in the zone where the wall thickness is equal to *e_s_* (see Figure 1b). The admissible stress *f* equals to *f_min_* if the material chosen for the cylindrical and flat parts of the boiler is the same, and *p_c_* is, as previously, the internal pressure. Constants *C_1_* and *C_2_* are taken from the respective plots presented in the code EN 13445-3.

Regarding EN 12952:3, the following set of Equation (3) is used for the evaluation of the groove parameters.

(3) $$\{\begin{array}{c} e_{h1}\geq e_{s}\\ e_{h1}+r_{ik}\leq e_{h}\\ r_{ik}\geq \max\left\{\begin{array}{cc} 0.2\cdot e_{s}, & 5 \end{array}\right\}\\ e_{h1}\geq 1.3\left(\frac{d_{i}}{2}-r_{ik}\right)\cdot \frac{p_{c}}{f} \end{array},$$

where *e_s_* is the shell wall thickness and *d_i_* the inner boiler diameter, *p_c_* is the internal pressure, and *f* the admissible stress. The reported set of inequalities defines certain ranges for the groove radius *r_ik_* and the minimum endplate thickness *e_h1_*. The obtained admissible values for pairs (*r_ik_*, *e_h1_*) usually cover certain polygonal areas. When the code EN 13445-3 is used, the set of respective conditions is different.

(4) $$\{\begin{array}{c} e_{h1}\geq e_{s}\\ e_{h1}+r_{d}\leq e_{h}\\ r_{d}\geq \max\left\{\begin{array}{cc} 0.25\cdot e_{s}, & 5 \end{array}\right\} \end{array}.$$

The main difference with the approach defined by set (4) is in the introduction of locally increased wall thickness *e_s_* instead of the calculated wall thickness *e_p_* (Figure 1b). Additionally, the fourth condition included in set (3) is no longer present. Besides this, the additional formulae for the *l_cyl_*, i.e., the length of the reinforced cylindrical part, is introduced in the form:

(5) $$l_{cyl}=\sqrt{\left(d_{i}+e_{s}\right)\cdot e_{s}}.$$

The considered code also recommends that the transition zone in the cylindrical part, in which the wall thickness increases from *e_p_* to *e_s_*, should be smooth, with the inclination angle γ being not bigger than 30° (see Figure 1). Unfortunately, no clear suggestion is given in the code as to how to assume the thickness *e_s_* in the thickened area. This gives certain additional freedom in the designing process and opens up the possibility of the search for the optimal combination of the investigated structure parameters (dimensions) in the junction providing the minimum stress concentration. The set of inequalities presented in Equation (4) results in a triangular area in the (*r_d_*, *e_h1_*) coordinates, which limits the values for the lowest thickness in the groove and the radius of the groove. The latest experiments have shown that the inclination angle of the groove γ (Figure 1a) has no real influence on the stress concentration when its value exceeds 60° [9], so all the numerical calculations presented in the paper assume an angle value γ = 90°. In such a case, the problem of the search for optimal parameters is reduced to the determination of only two design variables when using the EN 12952:3 code, i.e., the minimum thickness of the endplate measured at the bottom of the groove, *e_h1_*, and the radius of the groove, *r_ik_* (see Figure 1a). When using the EN 13445-3 code, the additional, third design variables appear, i.e., thickness *e_s_* (see Figure 1b). The aforementioned problem of the search for optimal values for design variables that provide the minimum stress concentration can be regarded as parametric optimization, in which the optimal vector of the design variables is obtained on the basis of an analysis of the sequence of solutions obtained for different combinations of design variables. Different optimization criteria and optimization tools can be applied in the search for the optimal groove parameters [21,22]. The most common approach in structural problems is the definition of the objective function as the minimization of the maximum value of the stress concentration factor, which is expressed as follows:

(6) $$F_{s}=\min\left\{\max\frac{\sigma_{\mathrm{eqv}}}{\sigma_{Y}}\right\},$$

where *σ_Y_* is the yield limit, while σ_eqv_ defines the equivalent stress following the von Mises or Tresca-Guest hypothesis. This formula is fully justified for structures which exhibit only an elastic response to the applied load, but usually fails in the case of structures where elastic-plastic deformation may appear, particularly when the hardening modulus is rather small. In such a situation, the minimization of the maximum value of the equivalent plastic strain seems to be the most convenient:

(7) $$F_{e}=\min\left\{\max\varepsilon_{pl\_eqv}\right\},$$

where ε*_pl_eqv_* is the equivalent plastic strain.

In the case of the optimization process including only two design variables, the simple search method in the admissible design space is one possible solution method. This approach makes it possible to study the distribution of the objective function over the whole domain, and prevents getting stuck with the solution in the local minimum. The above approach can also be used in the case of three design variables, as in the case of using EN 13445-3 code. Here, for the set value of the shell thickness *e_s_*, the area of the search in the sense of the inequalities given in Equation (4) is found. As a consequence, the bounding values for *e_h1_* and *r_d_* become different for different values of *e_s_*. This means that the optimal search can be done for the established value of *e_s_*. As shown, an increase of *e_s_* reduces the inner diameter of the cylindrical part, namely *d_i_*, which, finally, causes a reduction of the endplate thickness *e_h_*, which reduces the limits of admissible changes for the groove radius and the thickness at the groove bottom.

## 3. Finite Element Modeling

### 3.1. Material 

The investigated structure was made by means of welding from the steel 16Mo3, which is a low alloy steel that is widely used in the construction of pressure appliances due to its low-grained structure, good mechanical properties, and stable behavior after welding and heat treatment processes [23]. Subsequently, the weld area was modeled as having the same mechanical properties as the remaining part of the structure, and it was assumed that the welded structure was free from residual stresses. The details of the chemical composition of the material used are given in Table 1.

The material properties were designated from the experimental tensile tests. The samples were cut in longitudinal (seven samples) and transverse (seven samples) directions from the cylindrical pipe. The investigated material exhibited elastic-plastic behavior with hardening. Slight anisotropy, caused by manufacturing by rolling, was also observed in the experimental tests. The longitudinal samples showed a slightly higher yield limit and slightly lower tensile strength than the transverse samples (Table 2). The lowest yield limit (*σ_Y_MIN_* = 274.1 MPa) was observed for one of the transverse samples (the corresponding tensile strength was equal to *σ_u_ =* 442.5 MPa). 

**Figure 2:**
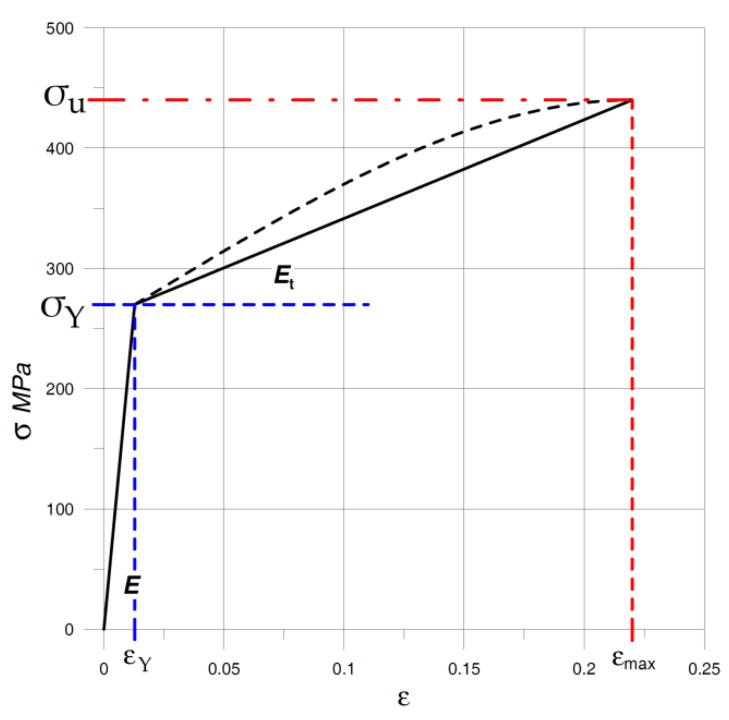
Real and bilinear stress-strain curve used in numerical calculations.

The numerical model of the material behavior was calibrated on the basis of the performed experimental tensile tests. Following the real stress-strain curve, the region of plastic deformation can be modeled as a nonlinear one, but to stay on the safe side, the linear hardening in the region of plastic deformations was used [24]. Finally, for calculation purposes, it was assumed that the Young modulus *E* = 2.1 × 10^5^ MPa, while the hardening module *E_t_* = 780 MPa, and the elastic Yield limit *σ_Y_* = 270 MPa and the tensile strength *σ_u_* = 440 MPa, and the maximum strain *ε_max_* = 0.22. A comparison of the real tension test curve and the assumed numerical analysis stress-strain curve is shown in Figure 2. 

### 3.2. Numerical Model

**Figure 3:**
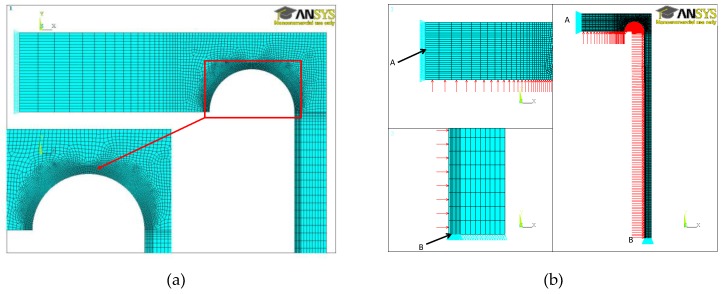
Finite element model: (**a**) part of the mesh in the flat endplate; (**b**) load—internal pressure and boundary conditions along edges A and B.

An analytical solution of the cylindrical shell–flat endplate junction subjected to internal pressure does not exist, so only certain assessments with various simplifying assumptions can be analytically undertaken. The results of such considerations are presented, for example, in papers by Kiesewetter [25] or Schwaigerer [26]. The lack of an analytical solution can be compensated for by using the numerical simulation and the finite element method, in particular. Here, ANSYS version 13 was applied in calculations. The all numerical analyses were performed with the use of the APDL facilities. Such an approach makes it possible to keep the same mesh densities in further optimization simulations. In this code, various structural problems, including material and geometrical nonlinearities can be solved. The analyzed structure, i.e., the pressure vessel with flat ends with a stress relief groove, exhibits axisymmetric properties of geometry and loadings, which means that only the symmetric quadrant of the axial cross-section can be considered in the analysis. Such a simplification allows for the application of relatively dense meshes of finite elements, particularly in regions where severe stress concentrations occur. The area of the stress relief groove is such a region. In order to receive reliable numerical results, the transition zone of small regular elements around the curved edge of the groove and its close neighborhood was introduced. The mesh generated in this subarea is adjusted with the change of the groove radii. Such an adjustment provides the demanded precision of the numerical calculations. Such a mesh generation helped us to keep the solution error under stress energy below 5% in each of the analyzed examples, which seems to be fully acceptable from an engineering point of view. Part of one of the used meshes is shown in Figure 3a with the groove, a curved area, magnified in the left bottom quadrant of the plot. In Figure 3b, the full analyzed numerical model is shown with the applied boundary conditions and with the internal pressure being applied as the load. Here, along edge A, the horizontal (radial, in the case of an axisymmetric structure) displacements are blocked, and along edge B, the vertical (along the vessel axis) displacements are blocked. The ANSYS PLANE82 plane finite elements with the axisymmetric option activated were used in the calculations. This finite element is the higher order eight- or six-node plane element with quadratic displacement approximation. This element is well suited for irregular meshes and analyses of structural problems with plasticity, creep large deflections, or large strains.

The applied technique of numerical modeling of the vessels with flat ends was experimentally verified for two kinds of stress relief grooves calculated with respect to the Standard EN 12952:3. The numerically-calculated and experimentally-measured distributions of strains on the outer surface of the vessel were compared. The strain gauges were placed in the area corresponding to the stress relief groove existing in the flat end [12]. The good agreement between both results (more details in [12]) allows for the assumption that the proposed numerical model is reliable for further analysis, as presented in the paper.

## 4. Results of Parametric Optimization

### 4.1. Stress Relief Groove Parameters Optimization Following the STANDARD EN 13445-3

**Figure 4:**
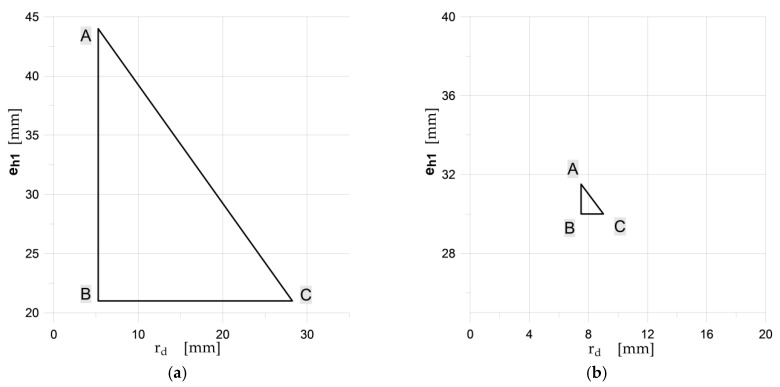
Admissible search area for optimal parameters for the radius of the groove and the minimum endplate thickness in the groove: (**a**) *e_s_* = 21 mm; (**b**) *e_s_* = 30 mm.

The detailed results presented below were obtained for a cylindrical shell following the shape presented in EN 13445-3 with an external diameter of ϕ406.4 and a basic wall thickness *e_p_* = 20 mm. In the study, the thickness *e_s_* was gradually increased from 20.0 mm to 30.0 mm, with a reasonable step of 1 mm (see Figure 1b). It appeared that for e_s_ = 31 mm, the admissible area for the minimum thickness at the groove bottom and the radius of the groove became empty, i.e., there were no admissible values fulfilling the set of inequalities (4). Table 3 shows the sets of variables used in calculations depending on the increased thickness *e_s_*. Figure 4 illustrates the admissible areas for the (*e_h1_*, *r_d_*) pairs obtained for the two thicknesses of the reinforced cylindrical shell part (thickness *e_s_*).

**Figure 5:**
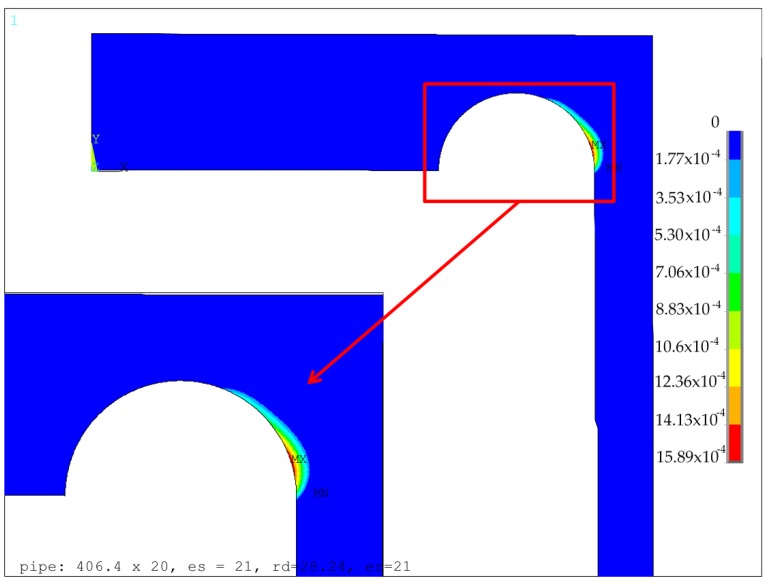
Distribution of equivalent von Mises plastic strains for the optimal parameters of the radius of the groove thickness in the thickened part of tube, *e_s_* = 21 mm.

**Figure 6:**
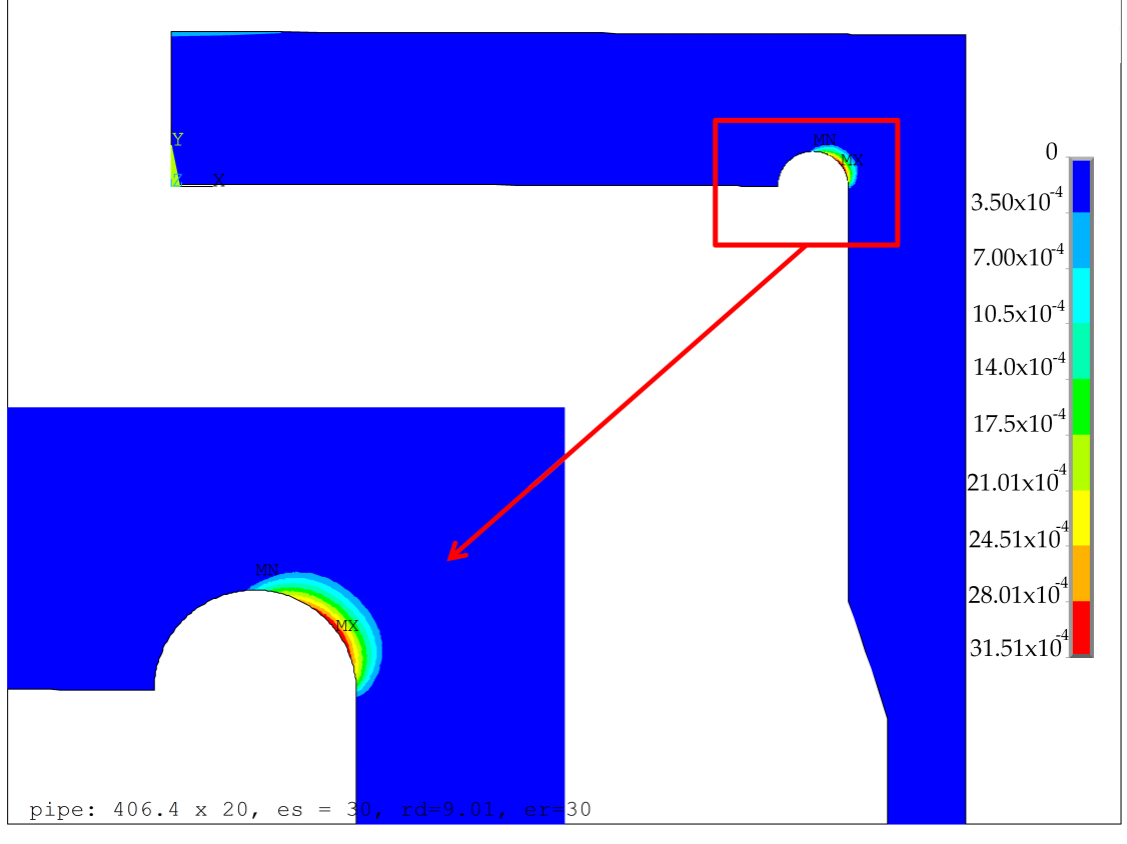
Distribution of equivalent von Mises plastic strains for optimal parameters of the radius of the groove thickness in the thickened part of tube *e_s_* = 30 mm.

**Figure 7:**
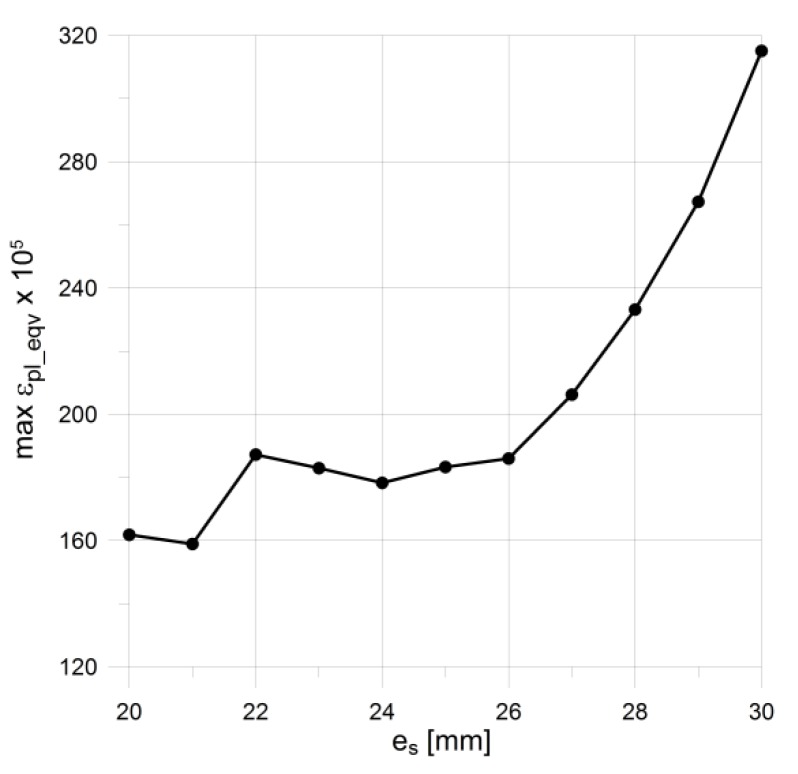
Maximum equivalent von Mises plastic strain distribution for the optimal radius of the stress relief groove with respect to the thickness of the reinforced part of the cylindrical shell *e_s._*

The optimization process and optimized parameters using the EN 12953:3 code were presented in papers [9,13]. The performed numerical calculations for the elastic-plastic material exemplified the presence of the point, where the minimum of the equivalent plastic strains appeared. For all the analyzed cases, the optimal point appeared on the borderline of the admissible area, where e_h1_ + r_d_ = e_h_, the location of the optimal point was rather close to the maximum admissible value of the groove radius. When using the code EN 13445-3, if only e_s_ is greater than 20.0 mm, then the optimal solution appears in the corner point C of the admitted area, which corresponds to the maximum admissible value of the groove radius. The exemplary results for the optimal configurations of the groove radii *r_d_* and the minimum thickness of the endplate *e_h1_*, obtained for two different values of *e_s_*, namely 21.0 mm and 30.0 mm, are presented in Figure 5 and Figure 6, respectively. These are the distributions of the equivalent von Mises plastic strains in the vicinity of the endplate-shell junction. The obtained results clearly showed that the maximum values of plastic strains were much more sensitive to the geometry changes (namely change of *e_s_*) than the values of equivalent stresses. This justifies the application of the objective function in the form given in Equation (7). Given the dependency between the maximum equivalent von Mises plastic strain obtained for the optimal parameters of the groove radii and the minimum endplate thickness presented in Figure 7, it is clearly seen that the minimum value was obtained for the shell with no local reinforcement being applied, namely *e_p_* = *e_s_*. This means that the introduction of the locally-thickened zone proposed in the EN 13445-3 code has the opposite effect to the expected one; it even deteriorates the structure resistance. Both codes used in the flat endplate calculations, namely EN 12952:3 and EN 13445-3, result in the presence of plastic deformations in the groove area which are completely unacceptable in applications where high cyclic loadings are applied and fatigue failure may appear. In such a case, the reduction of the applied operating pressure is the only remedy. However certain proposals of modifications of the structure’s topology improving the structure endurance are possible.

### 4.2. Increasing of Bending Stiffness of Flat End by External Rings

**Figure 8:**
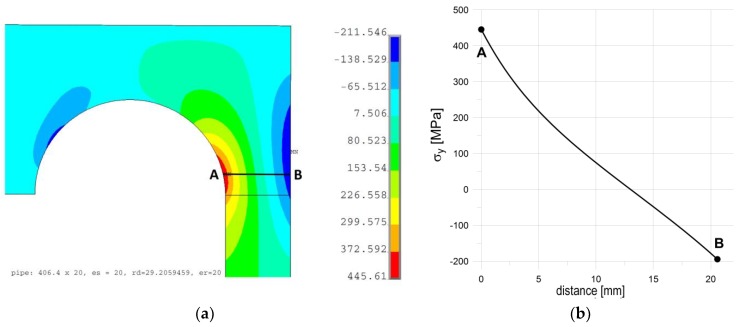
Illustration of the bending + tension effect in the axial cross-section of the analyzed vessel (purely elastic analysis): (**a**) Distribution of axial (meridional) stresses in the most strenuous part of the structure; (**b**) distribution of axial stresses across the pipe thickness.

**Figure 9:**
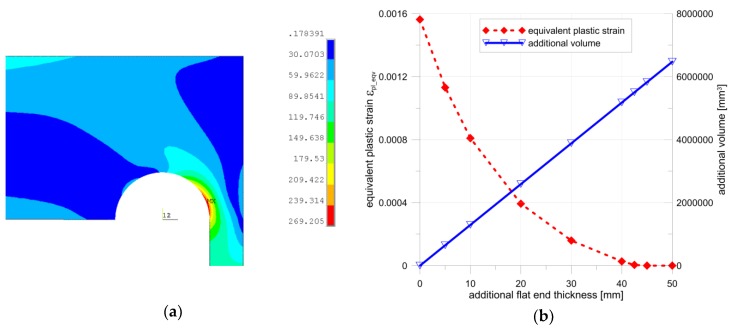
Structure with equally increased flat end thicknesses (additional thickness: 45 mm); (**a**) distribution of von Mises stress, (**b**) distribution of maximal equivalent plastic strains and additional volume value in mm^3^ with respect to additional flat end thickness.

If we apply the purely elastic model of the considered structure to the analysis, then a certain characteristic, structural behavior can be spotted. Detailed study of the maximal meridional (axial) stress shows that the axial stress in the tube, in the most strenuous cross-section, takes the characteristic course shown in Figure 8b. As shown, the axial stress varies almost linearly across the pipe wall; only a certain discrepancy (rapid increase) is observed inside the tube where stress concentration exists. In such a case, the resulting axial stress may be expressed in the form of the sum of the two components. The first one, i.e., the constant part, may be attributed to axial movement of the flat end subjected to the pressure force, while the second one is the result of bending appearing in the flat end. While the stress attributed to the pressure force cannot be reduced without the reduction of the acting inner pressure, the normal stress corresponding to bending can be limited by adding some material to the endplate which locally increases the end-plate stiffness. The simplest remedy seems to be a uniform increase of the end-plate thickness. Such an investigation was performed, and the results of the numerical calculations are shown in Figure 9. These studies were performed with the optimal stress relief groove parameters (*r_d_* = 29.206 mm and *e_p_ = e_s_* = 20 mm). As shown, a full elimination of plastic deformations was achieved with an additional thickness about 45 mm, which means that the introductory thickness of the endplate, suggested by the codes, was almost doubled. Such a situation of course raises the question of the economic aspects of such a design, i.e., with extremely heavy and massive flat ends. In summary, it means that no simple remedy can be applied to the studied problem.

**Figure 10:**
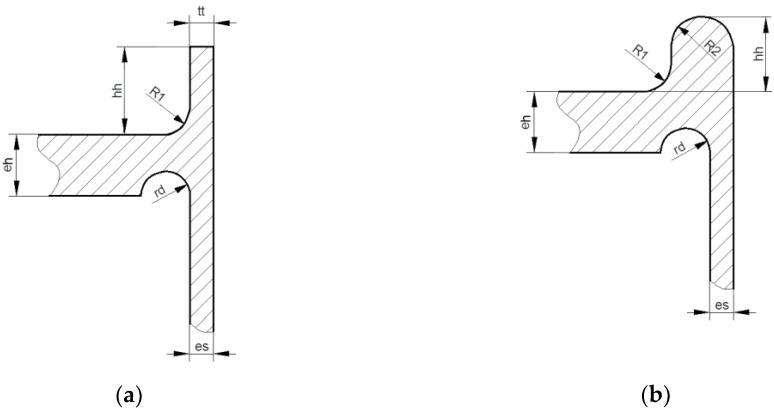
Two concepts of the proposed geometry modifications (part of axisymmetric model quadrant): (**a**) cylindrical ring around vessel circumference; (**b**) cylindrical rounded ring around vessel circumference.

The application of the biological growth approach proposed by Mattheck [27] may be useful in this case; this approach respectively adds or removes some material portions in the most and the least strenuous zones of structure. The engineering application of this approach has been presented in papers by Burchill, Heller, Waldman, and others [28,29]. Additionally, the proposed modification of the considered design should be low cost, as simple as possible, and should add or remove some parts of the material in a relatively easy way. Following these indications, some additional material is introduced on the outer side of the endplate, while the groove area remains unchanged. The idea of the proposed approach is illustrated in Figure 10. 

Here, the material is added in the form of a short cylindrical ring (Figure 10a), or in the form of a ring with a circular cross-section (Figure 10b). These two rings are placed and welded on the top of the endplate along its circumference. The preliminary numerical calculations performed for certain values for both proposals shown in Figure 10 were encouraging; it appeared that a full elimination of plastic deformations was possible for certain combinations of reinforcing ring dimensions and groove radii. Such observations encouraged the authors to perform detailed parametric numerical optimization with the objective function (6). With the application of a ring with a rectangular cross-section, the ring thickness (*tt*), height (*hh*), the transition radius (*R*1), and the groove radius (*r_d_*) were assumed to conform to the design variables. In order to simplify the study, the height of the additional ring *hh*, the thickness of the ring *tt*, and the transition radius *R*1 are expressed by means of three coefficients *k*_1_, *k*_2_, and *k*_3_, with respect to the cylinder thickness *e_s_* as follow:

(8) $$\{\begin{array}{c} hh=k_{1}\cdot e_{s}\\ tt=k_{3}\cdot e_{s}\\ R1=k_{2}\cdot k_{1}\cdot e_{s} \end{array}.$$

The choice of the values for coefficients *k*_1_, *k*_2_, and *k*_3_ was justified by the possibility of the use of finished goods which are accessible on the market. Such an approach guarantees the minimum economical cost. On the basis of the preliminary study, it was assumed that the aforementioned coefficients could change within the ranges:

(9) $$\{\begin{array}{c} k_{1}\in \langle \begin{array}{cc} 0.8, & 5.25 \end{array}\rangle \\ k_{2}\in \langle \begin{array}{cc} 0.1, & 0.9 \end{array}\rangle \\ k_{3}\in \langle \begin{array}{cc} 0.8, & 1.5 \end{array}\rangle \end{array}.$$

The change of the structure shape, namely, of the flat endplate, requires the search for the optimal combinations of design parameters *k*_1_, *k*_2_, *k*_3_, and *r_d_*. Following the former results, it was assumed that the center of the groove was located on the bottom edge of the endplate. 

**Figure 11:**
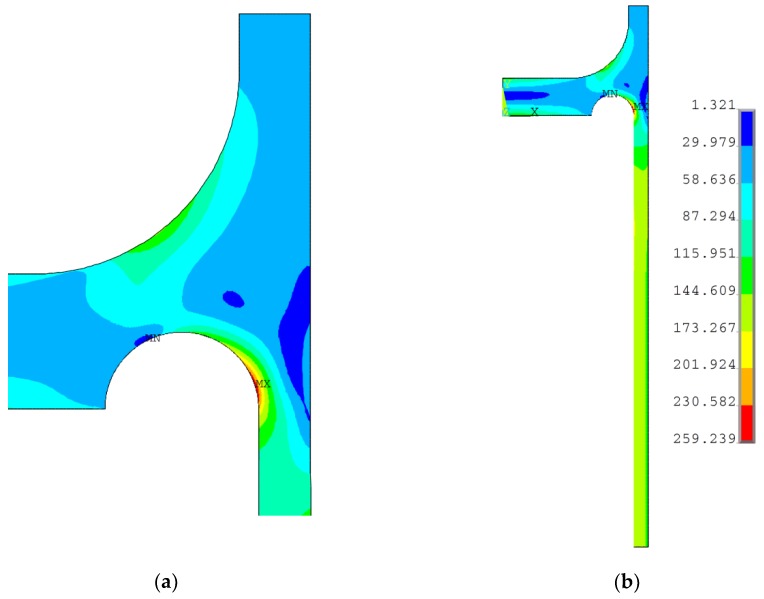
Distribution of von Mises stress in the flat end with a cylindrical rounded ring with parameters *e_s_* = 20.0 mm, *r_d_* = 29.760 mm, *k*_1_ = 5.033 *k*_2_ = 0.788, and *k*_3_ = 1.378: (**a**) area of the stress relief groove; (**b**) cross-section of the quarter-part of vessel.

**Figure 12:**
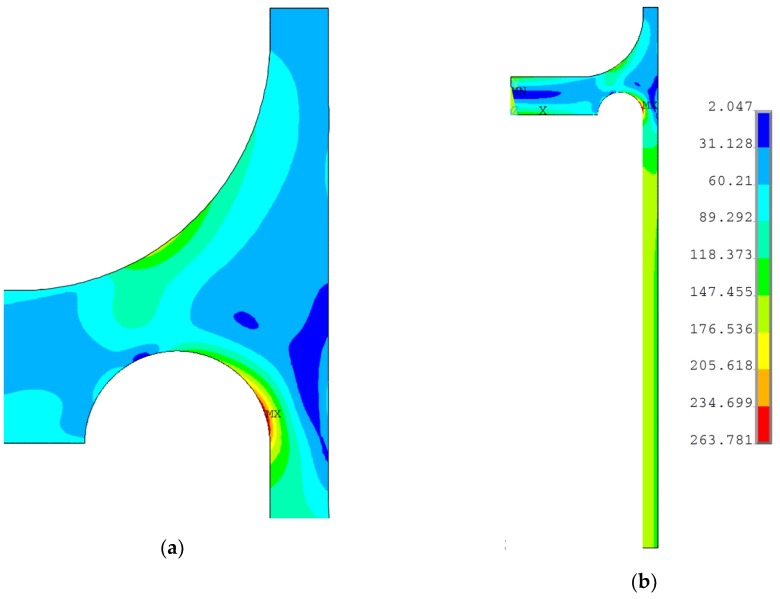
Distribution of von Mises stress in flat end with cylindrical rounded ring with parameters: *e_s_* = 20.0 mm, *r_d_* = 31.616 mm, *k*_1_ = 4.827 *k*_2_ = 0.891, and *k*_3_ = 1.0: (**a**) area of the stress relief groove; (**b**) cross-section of the quarter-part of vessel.

The numerical optimization problem was investigated by means of the two optimization methods, namely, the subproblem optimization procedure, which is the zero-order method, and the first order method, which also takes into account the gradient of the objective function [26,30]. The gradient method is much more time consuming and appeared to be less effective in the considered problem. Due to the observed relatively small changes/variations in the objective functions with respect to the design variable variations, certain difficulties with gradient calculations were encountered during the process, and problems with the solution convergence appeared. Finally, the results were obtained by means of the subproblem optimization method. The optimal results are as follows: *k_1_* = 5.033, *k_2_* = 0.788, *k_3_* = 1.378, *r_d_* = 29.76 mm, and the distribution of the equivalent stress for the optimal point is presented in Figure 11. As shown, the maximum equivalent stress value is below the yield limit, but still exceeds the allowable stress value, *f*. Additionally, the case, in which the reinforcing ring thickness *tt* has the same thickness as the cylindrical part *e_s_*, was studied (*k*_3_ = 1.0). Such an assumption eliminates one of the design variables (*k*_3_) and seems to be reasonable for application from an economic and technological point of view. In this case, also the full elimination of the equivalent plastic strains was achieved for the following optimal values of the design variables: *k*_1_ = 4.827, *k*_2_ = 0.891, and *r_d_* = 31.616 mm. Figure 12 presents the distribution of equivalent von Mises stresses obtained for the aforementioned design variable values. As in the previous case, the maximum equivalent stress is below, but close to, the yield limit value.

A study was also performed for a rounded ring added to the top of the endplate (see Figure 10b); again, satisfying results were obtained by means of the subproblem optimization method. In this case, radii *R*1, transition radius *R*2, and groove radius *r_d_* were assumed as the design variables. As in the previous analysis, the values of the respective radii were defined by means of the coefficients *k*_1_ and *k*_2_, which define the design variables with respect to the shell thickness *e_s_* as follows:

(10) $$\{\begin{array}{c} R1=k_{1}\cdot k_{2}\cdot e_{s}\\ R2=k_{1}\cdot e_{s} \end{array}.$$

On the basis of the preliminary calculations, it was assumed that the aforementioned coefficients would change within the ranges given below:

(11) $$\{\begin{array}{c} k_{1}\in \langle \begin{array}{cc} 0.5, & 2.5 \end{array}\rangle \\ k_{2}\in \langle \begin{array}{cc} 0.1, & 0.9 \end{array}\rangle \end{array}.$$

**Figure 13:**
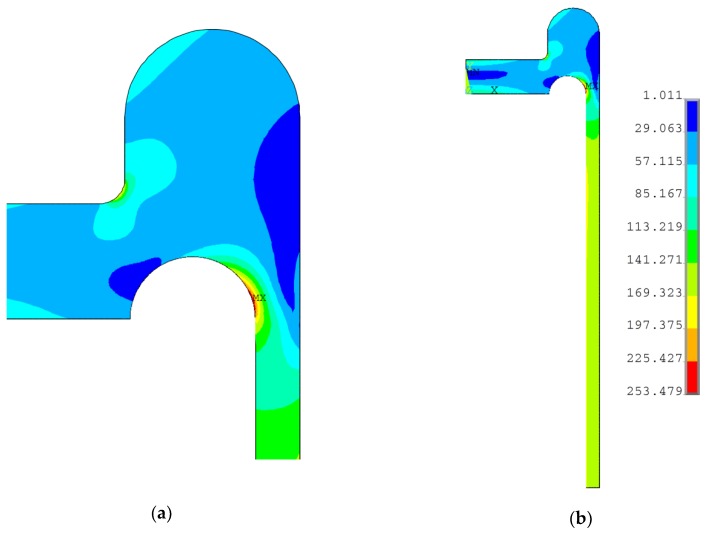
Distribution of von Mises stress in flat end with cylindrical rounded ring with parameters: *e_s_* = 20.0 mm, *r_d_* = 28.249 mm, *k*_1_ = 1.9709, and *k*_2_ = 0.2839: (**a**) area of the stress relief groove; (**b**) cross-section of the quarter-part of vessel.

The optimal values of the design variables obtained in the optimization process were as follows: *k*_1_ = 1.9709, *k*_2_ = 0.2839, and *r_d_* = 28.249 mm; the equivalent stress distribution is shown in Figure 13. Again, the obtained maximum value of the equivalent stress was high and close to yield limit, but did not exceed this value, which means that the investigated structure was free from plastic deformations.

All the aforementioned results for the performed optimization show that the complete elimination of plastic deformations is possible. However, the maximum value of stress is still very high and demands a certain reduction in the operating pressure. This reduction would be smaller than for a structure with no reinforcing ring, even with optimally-chosen stress relief groove parameters. The performed study has shown that it is possible to introduce relatively simple modifications in the design of the flat end pressure vessel which provide total elimination of plastic deformations. 

### 4.3. Topological Optimization of the Stress Relief Groove

The application of topology optimization makes it possible to distribute the material of a body on the basis of selected criteria [26,31,32,33,34]. In this process, the global stiffness, natural frequency, etc. can be stated as objective criteria. These measures can be optimized with respect to the assumed constraints (volume or mass reduction). Recent techniques and FE solvers also offer more advanced topology optimization techniques, the most interesting of which seems to be the stress-based [35] and fatigue damage-based [36] topology optimization methods. Such methods, with the simultaneous implementation of the recent multiaxial high-cycle fatigue criteria [37,38], are broadly applicable to the optimization and construction of structures which are subjected to both static and fatigue loads. In the performed analyses, the maximization of the structure stiffness was assumed as the objective function to the volume constraint. The use of such a method in the study is associated with the limited possibilities of the software used (ANSYS 13 APDL). In such a situation, it should be noted that the stiffest design is not necessarily equivalent to the design with the optimal, i.e., the smallest, stress concentration. Because of this, the application of the stress-based topology optimization of pressure vessels will be the subject of a future study.

In the FE solver (ANSYS 13) [26], topology optimization can be performed with the use of structural static compliance (SSC). SSC determines the energy of the work done on the investigated structure by the external loads. The minimization of SSC is equivalent to the maximization of the structure static stiffness. For application in the FE code, the compliance function (SSC) can be defined as:

(12) $$SSC=\int_{V}f\;u\;dv+\int_{S}t\;u\;ds+\sum_{i}F_{i}u_{i},$$

where *f* is body forces, *u* is displacements, *t* is traction forces, *F_i_* is the point load on *i*-th node, *S* is the surface area, and *V* is the volume.

In the optimization of the topology, the volume of the base structure is removed. Such a reduction of volume leads to an increase of SSC (reduction of stiffness). The main goal of the performed analysis is to find the best distribution of the material of the structure in order to achieve the minimum SSC (the maximal static stiffness) value, subject to the assumed volume reduction. Such calculations are made with the application of the method, which is known as the Solid Isotropic Material with Penalization Method. This is undertaken by the introduction of the pseudo-densities *η_j_* for each finite element of the model. Such pseudo-densities can be defined as a fraction of the material density:

(13) ηj=ρjρ0,

where *ρ_j_* is the density of the considered *j*-th element and *ρ_0_* is the density of the base material.

The pseudo-densities are assumed as design variables in the topology optimization process. The stiffness, *K_j_*, of a particular element of the optimal structure is determined by the pseudo-densities *η_j_* using the penalization method in the following way:

(14) $$K_{j}={\left(\eta_{j}\right)}^{k}K_{0},\;k>1,$$

where *K_0_* is stiffness of the base material and *k* is the equivalent for penalization power, usually *k* = 3 [32,34].

The application of the above formulae leads to the two following cases: *K_j_* > 0, i.e., material exists, and *K_j_* = 0, i.e., no material existence. This is controlled by the values of the pseudo-densities *η_j_* (*η_j_* ∈ <0−1>) and penalization power *k*.

A mathematical model of the applied topology optimization can be summarized as follows:

(15) $$\{\begin{array}{c} Find\;\eta ={\left(\eta_{1},\eta_{2},...,\eta_{n}\right)}^{T}\\ Minimize\;SSC(\eta )=H_{j}{}^{T}u\\ Subject\;to:\{\begin{array}{c} V=\sum_{j=1}^{n}\eta_{j}v_{j}\leq V^{*}\\ \begin{array}{l} 0<\eta_{\min}\leq \eta_{j}\leq 1\\ H_{j}=K_{j}u \end{array} \end{array} \end{array},$$

where: V* is the amount of material at our disposal, *V* is volume after optimization, and *η_1_*, *η_2_*,… *η_j_* are the design variables.

Topological optimization of the analyzed flat ends was performed with the use of the aforementioned mathematical model and the ANSYS 13 software. As observed in the previous section, in order to reduce the equivalent stress and to eliminate plastic strains, it was necessary to add some portions of material outside the area of the flat end. In the previous section, certain modifications of the structure followed the common engineering practice that was presented and discussed. However, the question still remains as to whether the reduction of the stress concentration can be achieved using a more optimal distribution of the added material. Such a problem was solved by means of applied topological optimization. Different geometries of the flat end with added material have been used as the initial starting models. In all the performed studies, the material was added only outside the structure. This was caused by the limitations included in the respective Standards [10,11]. The topology optimizations were made with the application of the Optimality Criteria Method (OC) and the Sequential Convex Programming approach (SCP). Only the most reasonable and optimal solutions are presented in the following section.

**Figure 14:**
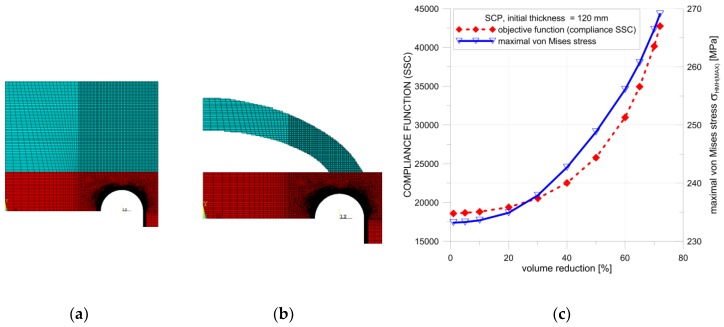
Topology optimization, geometry 1, SCP method: (**a**) starting structure; (**b**) optimized structure with 72% volume reduction; (**c**) distributions of SSC and von Mises stress with respect to the volume of the structure.

**Figure 15:**
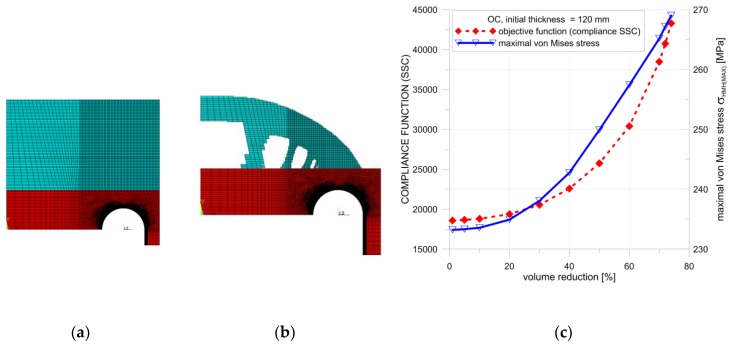
Topology optimization, geometry 1, OC method: (**a**) starting structure; (**b**) optimized structure with 74% volume reduction; (**c**) distributions of SSC and von Mises stress with respect to The volume of the structure.

Several calculation attempts were performed; below, the results of only two studies with different material arrangements are presented. A common feature of both proposals was the introduction of the additional material only outside the vessel, in the vicinity of the area where the maximum stress concentration was observed. Only this added material was subjected to the topology optimization, due to the conditions given in Standards [10,11]. In the first example, the thickness of the flat end was uniformly increased. The optimal solution (Figure 14b and Figure 15b) was obtained from a structure with an increased initial thickness of the endplate set to 120 mm (Figure 14a). The obtained maximal possible reduction of the added volume was equal to 72% for SCP (Figure 14b) and 74% for the OC method (Figure 15b). In both cases, the von Mises stress was slightly lower than the yield stress.

Detailed calculations of the topological optimization were performed with the structure volume reduction, which was reduced with a 5% step. The SSC and von Mises stress values were kept under control during this process. In each step, the structure with the maximum volume reduction was considered optimal only if the von Mises stress remained below the yield limit. The distribution of the controlled SSC process and the maximum von Mises stress values is illustrated in Figure 14c. The respective calculations were performed for both methods in ANSYS, namely, the SCP and OC methods. In both considered cases, small differences in the final volume reductions were observed. The main difference in the structure geometry was observed in the results of the OC method. In the optimal structure, additional ribs were present between the curvilinear part and flat end using this approach.

**Figure 16:**
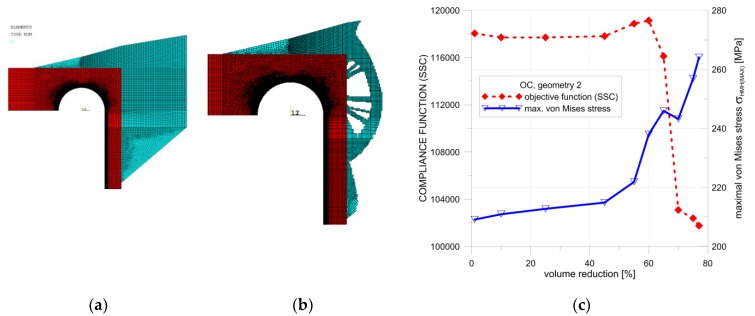
Topology optimization, geometry 2, OC method: (**a**) starting structure; (**b**) optimized structure with 77% volume reduction; (**c**) distributions of SSC and von Mises stress with respect to the volume of the structure.

**Figure 17:**
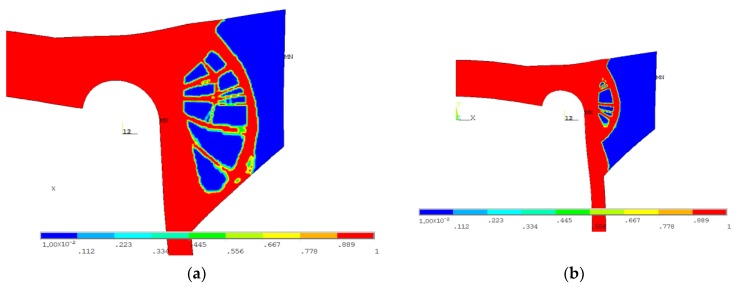
Distribution of averaged nodal pseudo-densities, geometry 2, OC method: (**a**) optimized structure with 60% volume reduction; (**b**) optimized structure with a 77% volume reduction.

**Figure 18:**
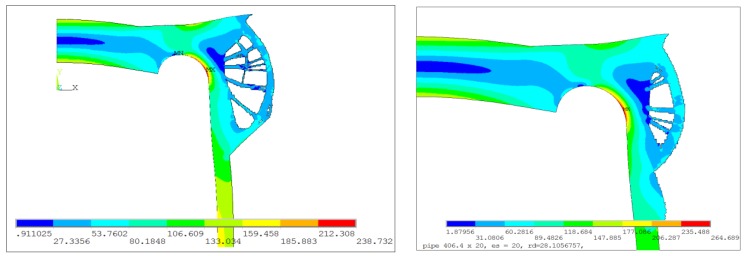
Distribution of von Mises stress, geometry 2, OC method: (**a**) optimized structure with 60% volume reduction; (**b**) optimized structure with a 77% volume reduction.

The second presented proposal relied on the introduction of additional material mainly around the top part of the cylindrical pipe and the connection pipe–endplate. Also, some material, rigidifying the vessel bottom, was placed on the top of the endplate. Detailed calculations were performed with the use of the OC method, and the optimal configuration was obtained for the volume reduction using 77% of the added material (Figure 16b). The dependency between the SSC, the maximum von Mises stress, and the percentage of additional volume reduction are illustrated in Figure 16c. The surprising course of changes of the SSC parameter when crossing 60% was due to the change of the final geometry, as illustrated in Figure 17a. The final, optimal distribution of the additional volume is shown in Figure 17b; it corresponds to 77% of the additional volume reduction. Such a nonsmooth change in the structure geometry results in a rapid change in the SSC value when moving from 60% to 65% of the additional volume. The rapid change of the SSC has no influence on the maximum value of the von Mises stress. The distribution of the von Mises stress for 60% and 77% of the additional volume reduction are presented in Figure 18a,b. The geometry which was the result of the topological optimization, in this case, was rather complex and difficult to manufacture. Summarizing the first studied proposal, the model illustrated in Figure 14 and Figure 15 seems to be more convenient and better-suited to practical applications. 

### 4.4. Discussion

The design of pressure vessels implies optimal shaping with respect to the stability and strength of the shell. Due to the application of such structures in petrochemical, chemical, as well as nuclear applications, the safe and reliable performance of pressure vessels is a very important engineering issue. The performed analyses revealed that the recommendations included in the standards [10,11] are not sufficient to optimally design the parameters of the endplate stress relief groove in flat endplates. Moreover, it was observed that significant plastic deformations may occur in the boundary area of the stress relief groove if nonoptimal values for the groove parameters are used in the design. The performed calculations revealed that the geometries of flat ends with a circular stress relief groove proposed in the standards (EN 13445-3) allow only for a reduction (i.e., for an optimal combination of *r_d_* and *e_h1_*), but not a complete elimination, of the plastic strains. However, the presence of such plastic strains is unacceptable in many practical applications. The elimination of excessive elastic-plastic deformations in the groove area demands certain modifications in the structure design. On the basis of a series of numerical tests, simple, technologically-justified modifications were proposed (see Figure 10). Next the optimization of their dimensions was performed. From the proposed shapes of the reinforcing rings, a full elimination of plastic deformations was achieved when optimal values for design variables were set. However, the level of equivalent stress was still high in the groove area, which demanded the reduction of the operating pressure. Such a design modification and the obtained analysis results became the inspiration for a more general approach in which the topology optimization was applied. Here, two concepts were incorporated.

The first one relied on a uniform increase in the endplate thickness with added material being placed on the outside boundary of the endplate. The second one proposed an increase in the tube thickness in the junction area of the tube and the endplate. Most of the added material was placed outside the top part of the tube, but a certain amount of material was used to locally increase the endplate thickness in the vicinity of the outer diameter. Both proposals gave satisfactory results in terms of plastic deformation elimination. However, the von Mises stress still remained close to the yield limit. The reduction of the added volume values exceeded 70% in both cases. The optimal structure configurations after topology optimization were of a rather complex shape, including internal ribs. A comparison of the obtained optimal results is summarized in Table 4. The volume of the whole vessel was about 4.0 × 10^7^ mm^3^. The volumes of the single endplate with optimal configurations of the stress relief groove are given in Table 4. In order to compare the obtained solutions in the last column of Table 4, the increase in the weight of the optimal flat end with added material is also presented.

## 5. Conclusions and Further Study

The performed numerical investigations confirmed the presence of strong stress concentrations in the area of the groove. A comparative study of flat endplates with grooves was performed following the code EN 13445-3, used in parallel with EN 12952-3, in standard calculations of pressure appliances. The results of that analysis proved that the local increase of the thickness *e_s_* in the transition head-shell zone did not improve the structure’s endurance. Then, a boiler with *e_p_* = *e_s_* = 20 mm and optimal stress relief groove parameters was chosen for further study. 

On the basis of engineering practice, the two proposed geometric modifications (see Figure 10) were studied and optimized with respect to the introduced design variables (see Equations (8)–(11)). As a result, the successful elimination of plastic deformation was observed. However, the maximum stress was still on a high level. A more general approach to the structure geometry modification was presented in two proposals used in the optimization of the topology. In the consequence full elimination of plastic deformations in the groove area was achieved. Such an approach (topology optimization) gives rise to more convenient material distribution compared to simple parametrical optimization.

In all the considered structures with modified geometries, full elimination of plastic deformations was reached. On the basis of the performed analyses, as well as the obtained optimized structures, it can be concluded that topological optimization is the most useful in optimizing the structural geometry. 

Future investigations will concentrate on noncircular [21,22], easy to manufacture grooves, whose shapes will be determined by parametrical or stress-based topology optimization. One additional area of further investigations concerns the application of vessels with noncircular (elliptical or rectangular) cross-sections and with stress relief grooves. These are included in both calculation codes, namely, EN 12952:3 and EN 13445-3. In this case, the junction between the flat endplate and the shell is not the only zone where stress concentrations are observed. In such cases, bending of the shell walls appears, which depends on the length of the constructed vessel. This can also alternatively limit the maximum applied inner pressure. 

## Figures and Tables

**Table 1:** Chemical composition of the low alloy 16Mo3 steel.

Chemical Composition [%], Fe Balance										
16Mo3	**C**	**Cr**	**Cu**	**Mo**	**Mn**	**N**	**Ni**	**P**	**S**	**Si**
	0.12 ÷ 0.20	≤0.30	≤0.30	0.25 ÷ 0.35	0.40 ÷ 0.90	≤0.012	≤0.30	≤0.025	≤0.01	≤0.35

**Table 2:** Material properties of 16Mo3 steel (SD: standard deviation).

Material Properties				
	Tensile Strength *σ_u_* MPa	Yield Limit *σ_Y_* MPa	Z %	A %
16Mo3 longitudinal direction (7 samples)	441.6 (SD: 8.8)	299.3 (SD: 8.6)	74.9 (SD: 0.5)	36.8 (SD: 1.4)
16Mo3 transverse direction (7 samples)	449.1 (SD: 4.0)	284.2 (SD: 6.6)	72.3 (SD: 0.8)	33.6 (SD: 1.6)

**Table 3:** Design parameters: *e_s_*, *d_i_*, *l_cyl_*, *e_h_*, and design variables (*e_h1_*, *r_d_*) range.

*e_s_*	*d_i_*	*l_cyl_*	*e_h_*	*r_d_*		*e_h1_*	
				min	max	min	max
20.00	366.40	0.00	52.14	5.00	32.14	20.00	47.14
21.00	364.40	89.96	49.24	5.25	28.24	21.00	43.99
22.00	362.40	91.96	44.89	5.50	22.89	22.00	39.49
23.00	360.40	93.91	43.95	5.75	20.95	23.00	38.20
24.00	358.40	95.80	43.36	6.00	19.36	24.00	37.36
25.00	356.40	97.65	42.54	6.25	17.54	25.00	36.31
26.00	354.40	99.45	42.08	6.50	16.08	26.00	35.58
27.00	352.40	101.21	41.16	6.75	14.16	27.00	34.41
28.00	350.40	102.93	40.36	7.00	12.36	28.00	33.36
29.00	348.40	104.62	39.68	7.25	10.68	29.00	32.43
30.00	346.40	106.26	39.01	7.50	9.01	30.00	31.51
31.00	344.40	107.87	38.34	7.75	-	31.00	-

**Table 4:** Comparison of optimal designs parameters of flat ends with stress relief grooves.

Geometry	Parameters	$\sigma_{eqv}^{\max}\mathrm{MPa}$	ε*_pl_eqv_*	Initial Flat End Volume mm^3^	Increase of Flat End Weight
Modification of flat end geometry with respect to EN 13445-3	*e_s_* = 21 mm, *r_d_* = 28.24 mm, *e_p_* = 20 mm	271.80 (Figure 5)	158.9 × 10^–5^	5.50 × 10^6^	-
Modification of flat end geometry with respect to EN 13445-3	*e_s_* = 30 mm, *r_d_* = 9.01 mm, *e_p_* =20 mm	272.70 (Figure 6)	315.1 × 10^–5^	6.57 × 10^6^	-
Increase of flat end thickness	*e_s_* = 20 mm, *r_d_* = 28.11 mm, e_p_ = *45* mm	269.21 (Figure 9a)	0	5.50 × 10^6^	105%
cylindrical ring around vessel circumference	*e_s_* = 20 mm, *r_d_* = 29.760 mm, *k_1_* = 5.033, *k_2_* = 0.788, *k_3_* = 1.378	259.24 (Figure 11)	0	5.37 × 10^6^	85%
	*e_s_* = 20 mm, *r_d_* = 31.616 mm, *k_1_* = 4.827, *k_2_* = 0.891, *k_3_* = 1.0	263.78 (Figure 12)	0	5.21 × 10^6^	76%
cylindrical rounded ring around vessel circumference	*e_s_* = 20 mm, *r_d_* = 28.249 mm, *k_1_* = 1.9709, *k_2_* = 0.2839	253.48 (Figure 13)	0	5.49 × 10^6^	103%
TO, geometry 1	*e_s_* = 20 mm, *r_d_* = 28.11 mm, Added volume reduction 72%, SCP method	269.1 (Figure 14c)	0	5.50 × 10^6^	79%
	*e_s_* = 20 mm, *r_d_* = 28.11 mm, Added volume reduction 74%, OC method	269.0 (Figure 15c)	0	Ok 5.55	73%
TO, geometry 2	*e_s_* = 20 mm, *r_d_* = 28.11 mm, Added volume reduction 77%, OC method	264.7 (Figure 18b)	0	5.50 × 10^6^	76%
